# Survey of Doctors' Experience of Patients Using the Internet

**DOI:** 10.2196/jmir.4.1.e5

**Published:** 2002-03-31

**Authors:** Henry W. W Potts, Jeremy C Wyatt

**Affiliations:** ^1^Cancer Research UK London Psychosocial GroupGuy's, King's and St Thomas' School of MedicineLondonUK; ^2^Klinische Informatiekunde (KIK)Academic Medical CentreAmsterdamNetherlands

**Keywords:** Internet, information quality, attitude to computers, questionnaires, patient education

## Abstract

**Background:**

There have been many studies showing the variable quality of Internet health information and it has often been assumed that patients will blindly follow this and frequently come to harm. There have also been reports of problems for doctors and health services following patient Internet use, but their frequency has not been quantified. However, there have been no large, rigorous surveys of the perceptions of Internet-aware doctors about the actual benefits and harms to their patients of using the Internet.

**Objective:**

To describe Internet-literate doctors' experiences of their patients' use of the Internet and resulting benefits and problems.

**Methods:**

Online survey to a group of 800 Web-using doctors (members of a UK medical Internet service provider, Medix) in September and October 2001.

**Results:**

Responses were received from 748 (94%) doctors, including 375 general practitioners (50%). Respondents estimated that 1%-2% of their patients used the Internet for health information in the past month with no regional variation. Over two thirds of the doctors considered Internet health information to be usually (20%) or sometimes (48%) reliable; this was higher in those recently qualified. Twice as many reported patients experiencing benefits (85%; 95% confidence interval, 80%-90%) than problems (44%; 95% confidence interval, 37%-50%) from the Internet. Patients gaining actual physical benefits from Internet use were reported by 40% of respondents, while 8% reported physical harm. Patients' overall experiences with the Internet were judged excellent 1%, good 29%, neutral 62%, poor 9%, or bad <1%. Turning to the impact of patient Internet use on the doctors themselves, 13% reported no problems, 38% 1 problem, and 49% 2 or more problems. Conversely, 20% reported no benefits for themselves, 49% 1 benefit, and 21% 2 or more benefits.

**Conclusions:**

These doctors reported patient benefits from Internet use much more often than harms, but there were more problems than benefits for the doctors themselves. Reported estimates of patient Internet usage rates were low. Overall, this survey suggests that patients are deriving considerable benefits from using the Internet and that some of the claimed risks seem to have been exaggerated.

## Introduction

While predictions have been made [1], little is known about how patient use of the Internet currently affects frontline clinicians. High quality information on the Internet is assumed to be vital for patients. Poor quality information presents obvious risks, including self-mistreatment and misdiagnosis (which can lead in turn to mistreatment or unnecessary worry in the patient), but the misunderstanding or misinterpretation of high quality information is also a potential problem. Even high quality information used well can challenge clinicians, leading to increased patient demand for their time and services [2]. A common, disheartening scenario is that of the patient entering the doctor's consulting room laden with Internet printouts.

However, increased information can improve the patient's understanding of the patient's condition, self-care, and state of mind [3], or even educate the doctor [2,4]. The right information can avoid unnecessary consultations, yet ensure prompt help-seeking when needed - the rationale behind NHS Direct Online [5] in the UK. The Internet can also act as a medium for social support [6]. It is important to recognize that patients may not want the same kind of information as clinicians. For example, patients may wish to read other's autopathographies [7], narratives about another's experience of illness. Such texts may fare badly under the usual evidence-based criteria, but may provide the personal experience and reassurance desired.

The Internet is not only about exchanging information: it can also provide access to services, such as buying drugs and other health products. It remains unclear how harmful or beneficial such services may be [8]. The activity is currently largely unregulated [9] and the American Medical Association has warned of the dangers of online prescribing [10], which has become a popular route for obtaining sildenafil and, since the events of late 2001 in the US, the anthrax antibiotic, ciprofloxacin [11- 14.

To explore the range of benefits and problems that Internet use by patients produces for themselves and for health services, we conducted a survey through an Internet service provider exclusively for UK doctors. Although not a representative sample, as early adopters, such users are likely to be more familiar with the Internet themselves and, thus, more aware of their patients' Internet use. While this cannot be a definitive survey, it explores the range of benefits and problems seen with patient Internet use in order to guide future research.

We did not ask patients about their experiences, but only their doctors. By surveying doctors, we could concentrate on Internet use that has a palpable effect on the patient's health and for the health care system. However, we need to bear in mind that some patient Internet use will be obscure to the clinician. Moreover, respondents' views of patients' experiences will be filtered through their own perceptions. We suspect that doctors' responses to questions about benefits or harms from their patients accessing Internet health information will vary according to their personal attitudes to the Internet and their general willingness to share information with patients. We therefore included questions to explore these suspicions, implemented as questions the trustworthiness of Internet financial advice and views on patient leaflets.

## Methods

An anonymous questionnaire was presented via Medix [15], a free Internet service provider and Web portal available exclusively to UK General-Medical-Council -registered practitioners. At the time of the survey, Medix had about 9100 members, approximately 4% of GMC (General Medical Council) registrants. Medix is a commercial venture and carries out regular profit and not-for-profit survey research among members. Financial incentives are offered for responding to questionnaires but not for responding to specific questionnaires. Awards are given to Medix members using an algorithm that takes into account their having done questionnaires during a particular time period.

Two versions of the questionnaire were presented to any Medix member registered as practicing full- or part-time (based on information given at first registration). One version of the questionnaire (Appendix 1) asked about possible benefits of the Internet, the other (Appendix 2) about possible harms, with participants randomly assigned to one version by proprietary software. This was done to avoid framing effects (questions about negative effects biasing answers to later questions about positive effects, or vice versa) and to keep the questionnaire short. Background questions were included on both versions, as was an identical overall question about patients' experiences of the Internet. Some questions have not been analyzed in this paper. Respondents were not required to complete any fields on the questionnaires beyond their GMC number and password. Each version was presented to 400 doctors between September 27 and 3 October 3 2001 inclusive.

When Medix members log on to visit the Web site [15], they must give their GMC number and self-assigned password. Proprietary software checks this information and a list of available questionnaires. If the demographics of the member are suitable for an available questionnaire and the member has not already done or refused the questionnaire (either questionnaire in this case), the questionnaire is offered. The member can defer doing the questionnaire, refuse to do it, or do it. If the questionnaire is refused, the member is never asked about that questionnaire again. Responses are stored on a central database and proprietary software ensures, based on the GMC number, that multiple responses are not possible. All responses, rejections, and deferrals are date stamped and time stamped by the server on receipt.

Data were analyzed in SPSS for Windows 10.0.0 (SPSS Inc.). Confidence intervals for medians were calculated in Stata 5.0 (Stata Corporation) by bootstrapping. This involved calculating 999 simulated (bootstrap) samples from the empirical distribution function (see [16]).

## Results

### Quantitative Results

The questionnaire was answered by 748 doctors (374 for each version), a 94% response rate. Fifteen doctors said they did not see patients and are excluded from further analysis (10 doctors from the positively-framed questionnaire and 5 from the other questionnaire). On the key question of "Overall, how would you describe your patients' experiences with Internet health material?", a Mann-Whitney test showed no significant difference between respondents answering the positively- and negatively-framed versions of the questionnaire (U = 63815, *P*= .7, n = 719). Thus, responses to identical questions on both versions were combined.

Demographic data on the participants was available (with data missing on 2 doctors): gender (624 men, 107 women), year of qualification (median 1985, inter-quartile range 1979-1992), region (London 78, South East 91, South West 63, West Midlands 60, Eastern 50, Trent 54, North West 90, Northern and Yorkshire 71, Scotland 86, Wales 26, Northern Ireland 24, other 38; other was ignored in analyses by region) and specialty (general practice 375, medical 144, surgical 84, psychiatry 40, anesthetics and intensive therapy units 35, accident and emergency 17, radiology 15, other 21).

Gender and year of qualification of respondents were checked and found to be similar to the general Medix membership. Compared to all GMC registrants, Medix has a lower proportion of female members (who make up 30% of GMC registrants, where gender is known) and a higher proportion of members who qualified between 1970 and 1999. Medix members match (UK resident) GMC registrants on proportions split by the first letter of their postcode.

Asked to estimate the percentage of their patients accessing Internet health material during the last month (Table 1), the median response was 1%-2%. A 95% bootstrap percentile confidence interval covered the 1%-2% and 3%-5% categories. Doctors' estimates did not vary by region (Kruskal-Wallis chi-squared(10) = 6.2, *P*= .8), but did by specialty (Kruskal-Wallis chi-squared(7) = 32.0, *P*< .001), with general practitioners GPs (general practitioners) estimating the lowest figures (median 1%-2%) and surgeons the highest (median 3%-5%).

**Table 1:** Proportion of patients estimated to have accessed Internet health material in the last month - responses to question shown

**Category**	**Number**	**% of those who gave an estimate**
<1%	143	22%
1%-2%	191	30%
3%-5%	195	30%
6%-10%	83	13%
>10%	36	6%
Unsure	82	
Non-response	3	

Participants were asked what they think of the general quality of health information on the Internet. Responses were: 89, *don't know*; 128 *usually reliable*(20% of those who gave a judgement); 306, *sometimes reliable*(48%); 184, *sometimes unreliable*(29%); 24, *usually unreliable*(4%). The median was *sometimes reliable*(bootstrap confidence interval lies within that category). These data did not vary by region (Kruskal-Wallis chi-squared(10) = 8.1, *P*= .6) or specialty (Kruskal-Wallis chi-squared(7) = 8.2, *P*= .3). More-recently-qualified doctors rated information as more reliable (Spearman's correlation with year of qualification, *r*_S_= 0.14, *P*< .001, n = 641).

Asked on the same scale about the general quality of financial information on the Internet, many more (272) responded *don't know*. For those who made a judgement, 36% rated financial information as unreliable versus 32% rating health information as unreliable. On a Wilcoxon test, respondents were significantly more trusting of health information than of financial information ( *z*= 2.97, *P*= .003, n = 431). We also asked for respondents' judgement of the value of patient-information leaflets, such as those from Cancer BACUP [17]. Only 32 answered *not sure*. Of those who made a judgement, 90% rated them as *very useful* or *sometimes useful* rather than *neutral*, *sometimes harmful* or *often harmful*. The rating of Internet-health-information quality was significantly correlated with both that for Internet financial information quality ( *r*_S_= 0.16, *P*< .001) and the value of health information leaflets ( *r*_S_= 0.11, *P*= .004). The ratings of Internet financial-information and health-information leaflets were not significantly correlated ( *r*_S_= 0.02, *P*= .6).

Asked whether patients had experienced problems or benefits from using the Internet, many doctors answered *not sure*. However, among those who responded, there were many more reports of patients experiencing benefits than problems (Table 2). When prompted with specific examples, more respondents selected actual problems and benefits than on the earlier question (Table 3). Of the respondents: 184 (50%) did not report any problems for their patients and 108 (29%) reported 2 or more problems; 97 (27%) did not report any benefits for their patients and 186 (51%) reported 2 or more benefits. The problems and benefits were matched to allow comparison. Overall, benefits outweigh problems, although different aspects emerge on each list. The Internet was seen as being valuable for informing, advising, and providing support for patients about their condition. However, becoming misinformed about one's condition was also the most-selected problem.

**Table 2:** Proportion of patients estimated to have had health problems or benefits from Internet use - responses to questions shown

**Problems**	**Number**	**% (95% CI)**	**Benefits**	**Number**	**% (95% CI)**
Yes	92	44% (37%-50%)	Yes	160	85% (80%-90%)
No	119	56%	No	28	15%
Not sure	158		Not sure	180	
Non-response	0		3		

**Table 3:** Perceived health problems or benefits for patients from Internet use - responses to questions shown

	**Number**	**%**	**95% CI**
Ordering dangerous or ineffective drugs or other health products	45	12%	9-16%
Getting misleading second opinions from (purported) practitioners	63	17%	13%-21%
Getting misleading risk estimates	46	13%	9%-16%
Getting misleading advice from patient support sites	68	18%	15%-23%
Seeking appropriate medical help later	20	5%	3%-8%
Becoming misinformed about their condition	94	26%	21%-30%
Spending a pathological amount of time on the Internet - "Internet addiction"	41	11%	8%-15%
Other	29	8%	5%-11%

**Table 4:** Perceived physical harm or benefit to patients from internet use - responses to questions shown

**Harm**	**Number**	**%**	**Benefit**	**Number**	**%**
No	176	92%	No	65	61%
Slight injury or pain	4	2%	Slight benefit	23	22%
Mild injury or pain	6	3%	Mild benefit	14	13%
Serious injury	5	3%	Dramatic benefit	5	5%
Not sure	175		Not sure	256	
Non-response	3		Non-response	1	

There was again considerable uncertainty in responses to the questions on physical harm or benefit arising from Internet use, more so for the question about physical benefits (Table 4). Of those who gave an answer, only 8% reported actual harm having occurred, whereas 40% reported benefits. The common benefits from the Internet, like improved patient self-confidence, may seem less dramatic than the potential hazards, yet at every level of severity, benefits were more frequently reported.

**Table 5:** Perceived health problems or benefits for doctors and the health service from internet use - responses to questions shown

	**Number**	**%**	**95% CI**
Patients are less able to cope with their symptoms or disease	38	10%	7%-14%
Longer consultations	236	64%	59%-69%
Patients are less confident about self-care	34	9%	6%-13%
Patients not seeking medical help when it was needed	23	6%	4%-9%
Patients are coming in later for necessary investigation or treatment	9	2%	1%-5%
More unnecessary investigations	162	44%	39%-49%
More unnecessary treatments	81	22%	18%-27%
Other	50	14%	10%-17%

Participants were asked to describe overall their patients' experiences with Internet health material. The responses were: 5, *excellent*(1%); 204, *good*(28%); 452, *neutral*(62%); 66, *poor*(9%); and 1, *bad*(0%). The median response was neutral (bootstrap confidence interval lies within that category). The doctors' overall rating of patients' experiences with Internet health material was significantly correlated with their rating of Internet health information quality. However, the correlation was not especially large ( *r*_S_= 0.30, *P*< .001, n = 718). These data did not significantly vary by region (Kruskal-Wallis chi-squared(10) = 7.7, *P*= .7) or by specialty (Kruskal-Wallis chi-squared(7) = 13.0, *P*= .07).

Asked about problems for themselves and for the health service (Table 5), 47 (13%) did not report any problems for themselves and the health service and 181 (49%) reported 2 or more problems. 74 (20%) did not report any benefits and 113 (21%) reported 2 or more benefits.

### Qualitative Results

Respondents could give free-text responses under the Other heading for the questions on specific problems or benefits. They were also able to comment on the questionnaire as a whole. Certain themes emerged. Respondents recognized the value of the Internet in providing information, which could lead to more productive consultations. However, these also tended to be longer, a luxury not always available. Problems were often not with the information per se, but for the patient (and the clinician) to be able to sift through and evaluate the information.

Particular problems raised were patients' desire for new, generally-unavailable treatments: a cult of the new, engendered by our technophile society? Many other problems focused on alternative therapies. Respondents commented about how patients can put too much faith in the Internet and that this can undermine faith in the doctor, although it could also back up the doctor and improve confidence, a result seen in other research [18].

The Internet has no geographical boundaries, but it does have linguistic ones and US sites dominate the English-speaking Internet. UK patients, unused to the nature of the US health care system, may be especially vulnerable to the direct advertising of health care services. Concern was expressed in our survey that, unlike US patients, UK patients may be less likely to bear in mind commercial biases in information presented. Other problems concerning the unsuitability of advice written from within the US context were also reported.

Two particular diseases were mentioned often in connection with problems: multiple sclerosis and chronic fatigue syndrome. It is not surprising that chronic, debilitating diseases with limited treatment options, often affecting a young population, should be highlighted. The Internet's value when dealing with rare diseases was also highlighted. The ability of the Internet to bring together, from all around the world, patients with rare diseases and experts on rare diseases is significant [4.

In terms of serious health problems from using the Internet, 3 actual deaths were described: an accidental overdose of Viagra ordered over the Internet, and 2 delayed presentations of cancers after the patients had tried remedies found on the Internet. A fourth comment was ambiguous about whether a fatality occurred from a purposeful overdose performed based on information on how to do it from the Internet, a concern raised previously [19-20].

## Discussion

Overall, our survey paints a fairly-rosy picture of patient Internet use, although it is notable that respondents are only aware of a surprisingly-small proportion of their patients using the Internet for health material. Many more benefits than problems for patients were reported. Information, advice, and social support were frequently-identified benefits of the Internet for the patient, although becoming misinformed was also the most commonly-reported problem for patients. Reports of problems and benefits for the doctor and the health service were more mixed. Confirming past research [2], over half our doctors reported longer consultations as a problem for the health service, while nearly half named unnecessary investigations. Improved coping and self-care were identified as the main benefits to the health service.

Debate rages about the frequency of adverse effects from Internet use [19,21,22]. Five of our respondents reported cases of serious injury, with comments describing 3 or possibly 4 deaths resulting from Internet use. With no time frame placed on the question, this represents the experience over many years of several hundred doctors, so we feel it represents a quite-low rate of severe events.

A survey of primary care staff in Glasgow [2 found that those under 40 were more likely to refer to the Internet for drug information. In this study, we found that more-recently qualified doctors considered health information on the Internet more reliable. It is not surprising that a younger generation of clinical staff is more comfortable using the Internet. Many respondents pointed out that their clientele were socially deprived and without net access. We must not overlook that the Internet may also exacerbate existing socioeconomic inequalities of health and that it may be less relevant to some groups [23].

Clearly, both benefits and problems exist with patients' use of the Internet. It is reassuring that these doctors see more benefits for patients, but that is not a reason to be complacent about the problems. Poor-quality information matters less if patients can effectively judge it so. High-quality information is less useful if patients are overwhelmed with its volume. The relationship between the quality of information on the Internet and patient experiences is not straightforward. There is plenty of scope for more detailed research in this area.

Many respondents felt unable to answer some of the questions. Of 732 respondents, 82 said they were unsure how many of their patients had been accessing Internet health information, while 89 said they did not know what the quality of health information on the Internet is like. While current research may help with the latter, with the former we note that patient Internet use can be obscure.

## Abbreviations

GMC: General Medical Council

## Multimedia Appendix 1

Questionnaire that asked about possible benefits of the Internet:

## Multimedia Appendix 2

Questionnaire that asked about possible harms of the Internet:
