# Supplementary material for: Survey of Doctors' Experience of Patients Using the Internet
Source: J Med Internet Res. 2002 Mar 31;4(1):e5. doi: 10.2196/jmir.4.1.e5 (PMC1761928; doi:10.2196/jmir.4.1.e5)
Supplement: Supplementary file 2 [file jmir_v4i1e5_app2.htm]

Patients using the Internet


## Patients using the Internet

This survey is being conducted by Professor Jeremy Wyatt to explore UK doctor�s experiences of patients using the Internet. It is hoped that this study will be presented in a special issue of the BMJ. Responding counts toward the Medix awards programme.

---

  
Your GMC and Medix password ensure that only you can respond and that you do so only once.

|  |  |  |  |
| --- | --- | --- | --- |
| GMC Number | Medix Password | | | | |

**---

1. About how many patients do you see in a month?**  
Do not see patients
  

<20
  

20-100
  

100-200
  

>200

**2. Estimate the percentage of your patients accessing health material on the Internet during the last month**  
<1%
  

1-2%
  

3-5%
  

6-10%
  

>10%
  

Unsure

**3. How many patient health internet sites have you looked at in the last month?**  
None
  

1-2
  

3-5
  

6-10
  

>10

**4. What do you think of the general quality of health information on the Internet?**  
Usually reliable
  

Sometimes reliable
  

Sometimes unreliable
  

Usually unreliable
  

Don't know

**5. Where would you recommend a patient with diabetes looks on the Internet for information about their condition?**  

**6. What do you think about the quality of financial advice on the Internet?**  
Usually reliable
  

Sometimes reliable
  

Sometimes unreliable
  

Usually unreliable
  

Don't know

**7. What statement best describes your feelings about giving patients information leaflets, such as those from Cancer BACUP as part of clinical practice:**  
Very useful
  

Sometimes useful
  

Neutral
  

Sometimes harmful
  

Often harmful
  

Not sure

**8. Have any of your patients experienced health problems as a result of accessing material on the Internet?**  
Yes
  

No
  

Not sure

**9. What kind of problems did your patients experience? [tick all that apply]**  
� Ordering dangerous or ineffective drugs or other health products
  

Getting misleading second opinions from (purported) practitioners
  

Getting misleading risk estimates
  

Getting misleading advice from patient support sites
  

Seeking appropriate medical help later
  

Becoming misinformed about their condition
  

Spending a pathological amount of time on the Internet - "Internet addiction"
  

Other
  
     


**10a. Have any of your patients ever experienced physical harm from taking or doing something they read about on the Internet?**  
Not sure
  

No
  

Slight injury or pain
  

Mild injury or pain
  

Serious injury

**10b. If harmed, what appeared to be the cause?**  


**11. In general, what have been the problems for you and the health service of your patients using the Internet? [tick all that apply]**  
Patients are less able to cope with their symptoms or disease
  

Longer consultations
  

Patients are less confident about self-care
  

Patients not seeking medical help when it was needed
  

Patients are coming in later for necessary investigation or treatment
  

More unnecessary investigations
  

More unnecessary treatments
  

Other
  
     


**12. Overall, how would you describe your patients� experiences with Internet health material?**  
Excellent
  

Good
  

Neutral
  

Poor
  

Bad

**13. Do you, your firm or your practice have an Internet site with health information or links for your patients?**  
Yes
  

No, but planning to
  

No, but would like one
  

No

**14. Comments about the subject or the questionnaire**

Many thanks for responding.
  
  
  

---

Q102.html by **Q** 27/09/2001 07:48:33 /  Report problems
